# Hepatic carcinoma-associated fibroblasts induce IDO-producing regulatory dendritic cells through IL-6-mediated STAT3 activation

**DOI:** 10.1038/oncsis.2016.7

**Published:** 2016-02-22

**Authors:** J-t Cheng, Y-n Deng, H-m Yi, G-y Wang, B-s Fu, W-j Chen, W Liu, Y Tai, Y-w Peng, Q Zhang

**Affiliations:** 1 Cell-gene Therapy Translational Medicine Research Center, The Third Affiliated Hospital of Sun Yat-sen University, Guangzhou, China; 2 Guangdong Provincial Key Laboratory of Liver Disease Research, Guangzhou, China; 3 Department of Hepatic Surgery, The Third Affiliated Hospital of Sun Yat-sen University, Guangzhou, China

## Abstract

Although carcinoma-associated fibroblasts (CAFs) in tumor microenvironments have a critical role in immune cell modulation, their effects on the generation of regulatory dendritic cells (DCs) are still unclear. In this study, we initially show that CAFs derived from hepatocellular carcinoma (HCC) tumors facilitate the generation of regulatory DCs, which are characterized by low expression of costimulatory molecules, high suppressive cytokines production and enhanced regulation of immune responses, including T-cell proliferation impairment and promotion of regulatory T-cell (Treg) expansion via indoleamine 2,3-dioxygenase (IDO) upregulation. Our findings also indicate that STAT3 activation in DCs, as mediated by CAF-derived interleukin (IL)-6, is essential to IDO production. Moreover, IDO inhibitor, STAT3 and IL-6 blocking antibodies can reverse this hepatic CAF-DC regulatory function. Therefore, our results provide new insights into the mechanisms by which CAFs induce tumor immune escape as well as a novel cancer immunotherapeutic approach (for example, targeting CAFs, IDO or IL-6).

## Introduction

Tumor progression is not only based on the transformation and proliferation of malignant cells but also depends on the tumor microenvironment functions. Tumor microenvironment consists of extracellular matrix and stroma cells, including carcinoma-associated fibroblasts (CAFs), tumor-infiltrating inflammatory cells and endothelial cells. In the past, most of the studies regarding immune escape mainly concentrated on the effects of tumor parenchymal cells on immune cells. Recently, however, the tumor microenvironment was also observed to contribute to tumor immunosuppression.

CAFs, also named tumor-associated fibroblasts, are marked by high expression of α-smooth muscle actin, fibroblast activation protein, fibroblast surface protein, vimentin and fibronectin as demonstrated in our previous study^1^ and are the dominant tumor microenvironment cell type. Recent studies have shown that CAFs support tumorigenesis and progression by promoting cancer cell proliferation^2^ and invasion.^3^ In addition, CAFs also showed a great ability to modulate the recruitment and functions of various tumor-associated immune cells in some studies, indicating that they might have an important role in tumor immune escape. In our previous study, CAFs derived from hepatocellular carcinoma (HCC) tumors inhibited natural killer cell functions, which was characterized by low cytotoxic molecule expression and impaired cytokine production, leading to decreased natural killer cell cytotoxic activity when incubated with K562 cells. In addition, this suppression was restored by indoleamine 2,3-dioxygenase (IDO) or/and PGE2 inhibitors.^4^ It was reported that macrophage recruitment into tumors was induced by CAF-produced CCL2^5^ and CXCL14^6^ or by CAF-derived extracellular matrix modification,^7^ which resulted in enhanced tumor metastasis. Su X *et al.*^8^ reported that CAFs in melanoma, mammary and colon cancer tumors were able to recruit or induce Th17 cells. Moreover, CAFs upregulate the production of regulatory T cells (Tregs) by secreting TGF-β in tumor microenvironments.^9^ Michael R. Nazareth *et al.*^10^ also found that CAFs from human non-small cell lung cancer had a suppressive effect upon tumor-associated T-cell activation and this suppressive effect could be abrogated by the blockade of B7H1 or B7DC. These findings demonstrate that CAFs have an important regulatory role in the functional status of various immune cells by complicated and multiple mechanisms in tumor microenvironments.

Dendritic cells (DCs) are essential in the activation of naïve T cells and initiate a powerful adaptive immunity response against infection and tumors. Nevertheless, several groups found that in patients with cancer there existed a sharp decrease in peripheral blood DC levels.^11, 12, 13, 14^ Moreover, in most tumors, there is a subset of regulatory DCs with a low capacity to promote T-cell proliferation and a high capacity to support immune tolerance.^15^ These regulatory DCs, which are characterized by immune-regulatory molecules and markers, express high levels of immunoregulatory cytokines or factors and induce Treg differentiation, thus helping tumor cells dodge immune defenses. At present, it is well known that tumor cells or tumor-derived factors are able to convert conventional or mDCs into regulatory DCs.^16, 17, 18, 19^ However, it is unclear whether non-tumor cells in tumor microenvironments can also induce regulatory DC differentiation. Given the notable regulatory effects of CAFs on various immune cells, we wondered about the role of CAFs in the generation of regulatory DCs.

HCC, which has an extremely poor prognosis, is one of the most common cancers throughout the world. Deciphering the immunosuppressive mechanisms that are induced by HCC and its microenvironment will help us better understand HCC pathogenesis and improve the immunotherapy strategies to combat it. In this study, we focused upon the capacity of hepatic carcinoma-associated fibroblasts (hCAF) to modulate the phenotype and functions of monocyte-derived DCs *in vitro* by using a cell-to-cell direct interaction model. We report here that hCAFs profoundly recruited DCs and converted them into IDO-producing regulatory DCs, which exhibited a tolerogenic phenotype with a remarkable ability to suppress T-cell proliferation and upregulate Treg production, through interleukin (IL)-6-mediated STAT3 activation. Therefore, our study initially showed that hCAFs have an immunosuppressive effect upon DCs, and provided us with a novel mechanism that involves tumor immune escape and a novel cancer immunotherapeutic approach (for example, by targeting CAFs, IDO or IL-6).

## Results

### hCAFs possessed the capacity to recruit normal DCs

**Figure 1:**
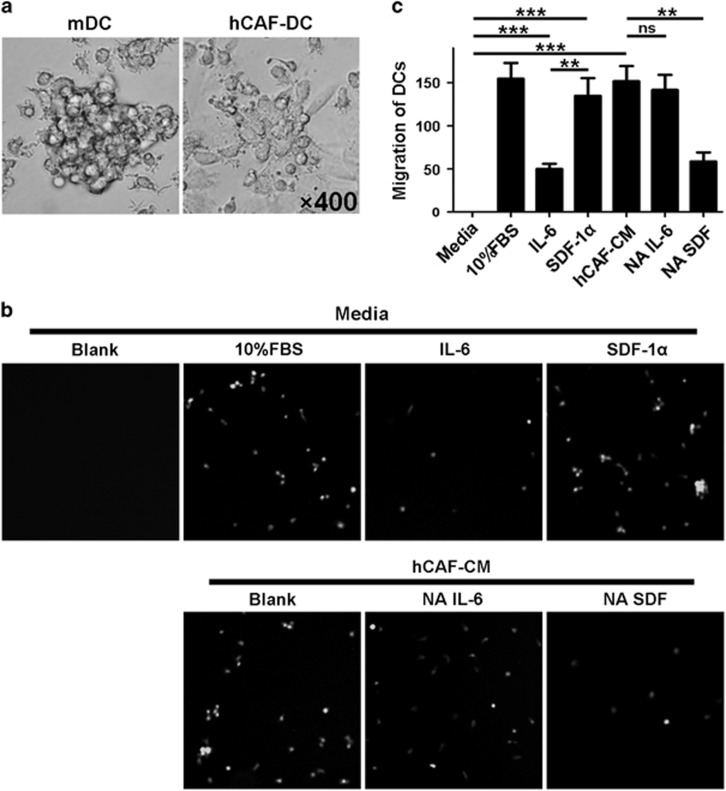
hCAFs possessed the capacity to recruit normal DCs. (**a**) The morphology of mDCs and hCAF-DCs was observed by inverted microscope ( × 400). (**b**) iDCs were purified and serum starved and then allowed to migrate for 3 h toward media alone (Blank), media with 10% FBS, media with IL-6, media with SDF-1α, hCAF-CM alone, hCAF-CM with NA IL-6 and hCAF-CM with NA SDF. Cell migration was observed by an inverted fluorescence microscope. (**c**) The data represent the mean ±s.e.m. of five different experiments. **P⩽0.01, ***P⩽0.001 and ns indicates non-significance.

To evaluate the potential immunoregulatory function of hCAFs, we set out to study their effects on DCs recruitment. As shown in Figure 1a, during co-culture DCs adhered to the hCAFs and aggregated vigorously. We also found that in contrast to normal mature DCs (mDCs), hCAF-co-cultured DCs showed fewer and shorter dendrites after lipopolysaccharide (LPS) treatment. Next we determined whether hCAFs were able to recruit normal DCs by using a transwell co-culture model. As a result, hCAFs possessed a strong capacity for DCs recruitment (Figures 1b and c). Stromal cell-derived factor (SDF)-1α is widely known as a potent chemokine produced from CAFs. As expected, when SDF-1α-neutralizing antibody (2 μg/ml) was added into hCAF-conditioned medium, migration of DCs was inhibited significantly. And IL-6-neutralizing antibody (2 μg/ml) had no effects on DCs migration. Furthermore, in normal media exogenous SDF-1α (50 ng/ml), but not IL-6 (50 ng/ml), could effectively recruit DCs. These results indicated that hCAFs are capable of recruiting normal DCs through an SDF-1α-dependent mechanism, which suggested that hCAFs might have potential immune-regulatory effects on DCs.

### hCAFs educated DCs to acquire a tolerogenic phenotype

**Figure 2:**
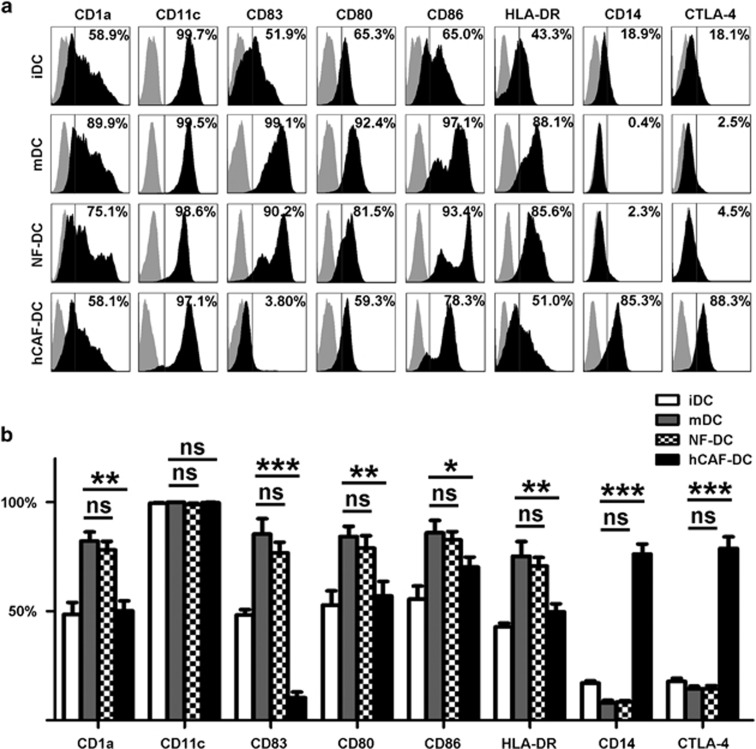
hCAFs educated DCs to acquire a tolerogenic phenotype. (**a**) Immunophenotype analysis of iDCs, mDCs, NF-DCs and hCAF-DCs was assessed by flow cytometry. One representative experiment of five is shown. (**b**) The data indicate the mean±s.e.m. of five different healthy subjects. The percentages of CD1a, CD11c, CD83, CD80, CD86, HLA-DR, CD14 or CTLA-4-positive cells are shown. *P⩽0.05, **P⩽0.01, ***P⩽0.001 and ns indicates non-significance.

Then, we further determined any DC phenotypic alterations by fluorescence-activated cell sorting after co-culturing with hCAFs or normal fibroblasts (NFs). As shown in Figure 2, hCAF-DCs expressed lower functional marker levels, such as antigen-presenting molecule CD1a, mature molecule CD83 and the costimulatory molecules, HLA-DR, CD80 and CD86 in contrast to the mDCs that were cultured alone. Unexpectedly, we found that hCAF-DCs also highly expressed CD14 and CTLA-4, which might be associated with their regulatory function.^15^ Moreover, there was no significant difference in the expression of these molecules between normal mature DCs and NF-DCs. For excluding the contamination of hCAFs, we also detected the human fibroblast markers CD90, CD166 and CD44 expression of the cells. And the expression of those molecules was negative (Supplementary Figure S1). These results showed that hCAFs were able to educate normal DCs to acquire a tolerogenic phenotype.

### hCAF-DCs exhibit tolerogenic characteristics

**Figure 3:**
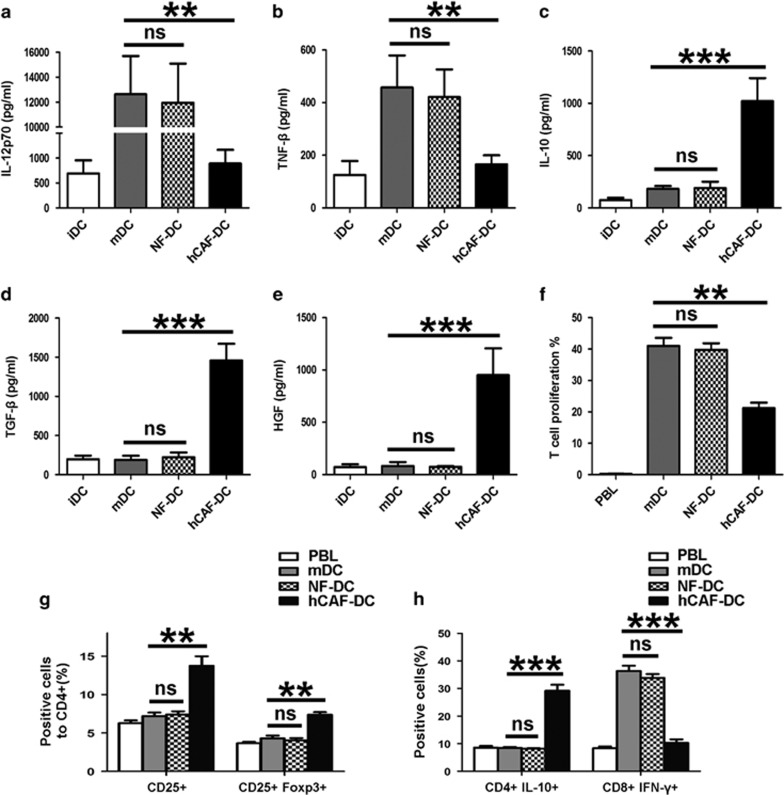
hCAF-DCs exhibit tolerogenic characteristics. (**a–e**) Cells supernatants were collected for IL-12p70, TNF-β, IL-10, TGF-β and HGF concentration measurements. The data indicate the mean±s.e.m. of five different experiments. (**f**) Proliferation of CFSE-labeled peripheral blood lymphocytes was determined by flow cytometry after culturing alone, or co-culturing with mDCs, NF-DCs or hCAF-DCs for 5 days. The data represent the mean ±s.e.m. of five different experiments. (**g–h**) Peripheral blood lymphocytes were cultured alone, or co-cultured with allogenic mDCs, NF-DCs or hCAF-DCs at a ratio of 5:1 for 5 days. Then, the cells were collected and examined by FACS. (**g**) The effect of mDCs, NF-DCs or hCAF-DCs on CD4+CD25+Foxp3+ Treg differentiation was assessed. The data indicate the mean±s.e.m. of five different experiments. (**h**) IL-10 expression in CD4+ T cell, and IFN-γ expression in CD8+ T cell was detected by intracellular staining. The data represent the mean±s.e.m. of five different experiments. **P⩽0.01, ***P⩽0.001 and ns indicates non-significance.

Considering that hCAF-DCs downregulated the expression of functional markers, we predicted that they might possess immunosuppressive functions. To further define the hCAF-DCs tolerogenic characteristics, we determined their cytokine profile and their ability to promote CD3^+^ T-cell proliferation. As a result, we found that in contrast to mature DCs, hCAF-DCs tended to express more immunosuppressive cytokines, such as IL-10, TGF-β and HGF, and less IL-12p70 and TNF-β (Figures 3a and e). We also evaluated the antigen-presenting ability of hCAF-DCs, and found that they had much less ability to stimulate carboxy fluoroscein succinimidyl ester-labeled CD3^+^ T-cell proliferation than mDCs (Figure 3f and Supplementary Figure S2A). Therefore, these data demonstrated that hCAF-DCs achieve functional immune tolerance characteristics.

Another important tolerogenic activity of regulatory DCs is inducing Treg differentiation and T-cell anergy.^20, 21^ Next, we assessed for functional T-cell changes after they were co-cultured with mDCs, NF-DCs or hCAF-DCs. Our findings showed that hCAF-DCs induced significantly higher percentage of CD4^+^CD25^+^Foxp3^+^ Tregs, compared with normal mDCs (Figure 3g and Supplementary Figure S2B). Intracellular staining further showed that IL-10 expression levels in CD4^+^ T cells were significantly increased, with the distinctly decreased IFN-γ levels in CD8^+^ T cells after hCAF-DC treatment compared with those co-cultured with normal DCs (Figure 3h and Supplementary Figure S2C).

Unlike hCAF-DCs, NF-DCs did not exhibit the same immunosuppressive characteristics (Figures 3a–h), which implied that those effects only occurred in co-cultivation with CAFs. These results indicated that hCAF-DCs, like regulatory DCs in tumors, had a potent ability to induce compromised T-cell responses, which proved that the observed hCAFs educated DCs to acquire an immunosuppressive capacity during immune reactions.

### hCAF-DCs induced compromised T-cell responses via IDO secretion

**Figure 4:**
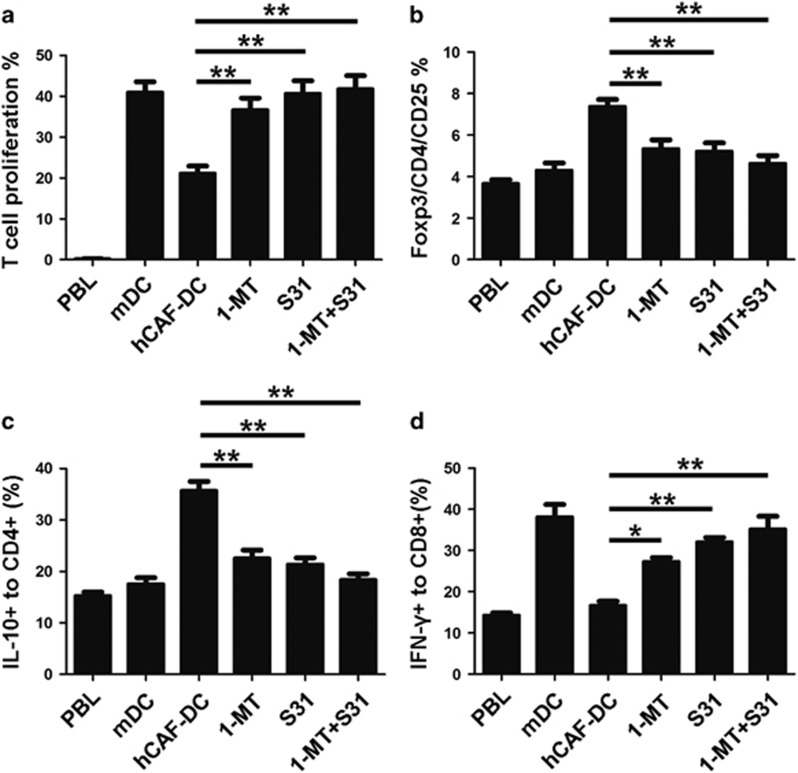
hCAF-DCs induced compromised T-cell responses via IDO secretion and STAT3 activation. hCAF-DCs were pre-treated with the STAT3 inhibitor S31–201 and/or the IDO inhibitor 1-MT for 1 day. Peripheral blood lymphocytes were co-cultured with mDCs or hCAF-DCs as described previously. (**a**) CFSE-labeled peripheral blood lymphocyte proliferation was determined by flow cytometry. The data indicate the mean±s.e.m. of five different experiments. (**b**) The CD4+CD25+Foxp3+ Treg percentages were analyzed by flow cytometry. The data indicate the mean±s.e.m. of five different experiments. IL-10 expression in CD4+ T cell (**c**) and IFN-γ expression in CD8+ T cell (**d**) was detected by intracellular staining. The data represent the mean±s.e.m. of five different experiments. *P⩽0.05, **P⩽0.01.

In the above study, we proved that hCAFs could impair DC functions and educate them to acquire an immunosuppressive capacity. In the following study, we focused on the mechanisms of how hCAF-DCs to modulate T-cell responses as well as how hCAFs to induce these regulatory DCs. IDO expression was considered as one of the main tolerogenic pathways in regulatory DCs.^22^ Therefore, we predicted that hCAF-DCs might alter T-cell responses through IDO-related pathways. As expected, when the IDO-specific inhibitor 1-methytryptophan (1-MT) was added into the mixed lymphocyte reaction (MLR) co-culture system, the immune suppressive effects were inhibited significantly. As shown in Figure 4a, 1-MT (0.2 μmol/ml) restored the capacity of hCAF-DCs to promote T-cell proliferation. We also found that once 1-MT was added, Treg induction and T-cell cytokine production could be reversed effectively (Figures 4b and d). Therefore, we proved that IDO has a vital role in the immunosuppressive effects of hCAF-DCs.

### IDO secretion in hCAF-DCs was dependent upon STAT3 activation

**Figure 5:**
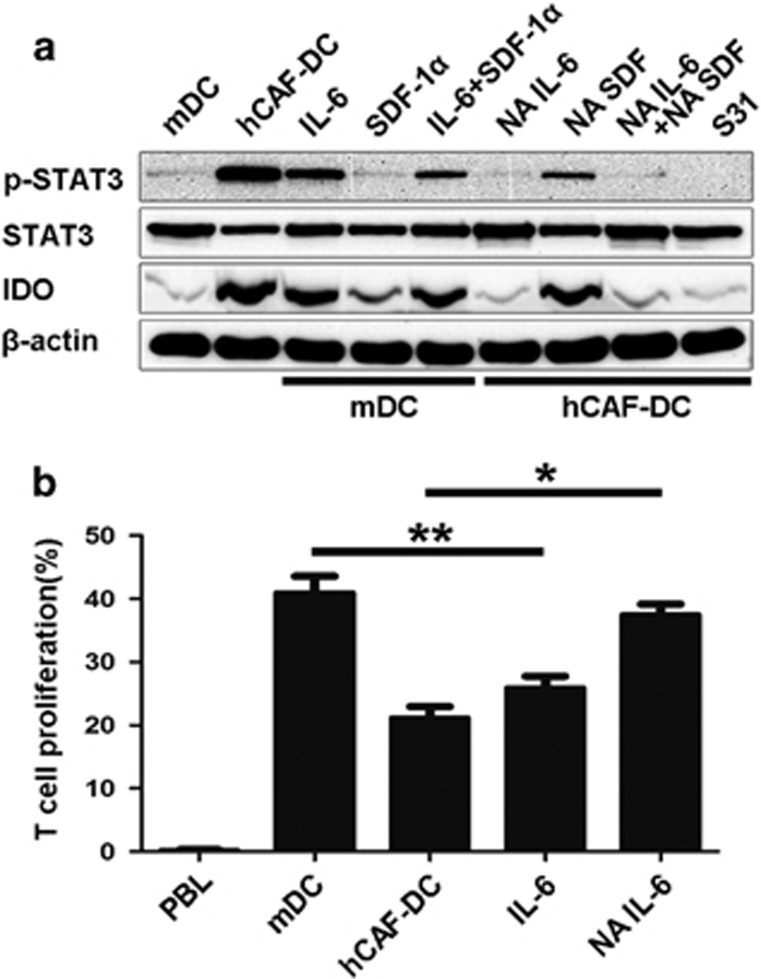
IL-6 is responsible for hCAF-DC STAT3 activation and IDO production. (**a**) STAT3 activation and IDO expression were analyzed by western blotting. (**b**) CFSE-labeled peripheral blood lymphocytes were cultured alone, or co-cultured with mDCs, hCAF-DCs, IL-6-treated mDCs and NA IL-6-treated hCAF-DCs. Peripheral blood lymphocyte proliferation was determined by flow cytometry. The data represent the mean ±s.e.m. of five different experiments. *P⩽0.05, **P⩽0.01.

It was reported that IDO may be induced by activation of STAT3 pathway.^23^ Tina L. Sumpter *et al.*^24^ also found that induction of IDO in myeloid DCs was dependent upon STAT3 activation. Therefore, we investigated whether the STAT3 pathway mediates IDO induction in hCAF-DCs. As expected, when treated with S31–201 (1 μl/ml), a STAT3-specific inhibitor, IDO production in hCAF-DCs was downregulated significantly (Figure 5a). Similarly, when STAT3 was inhibited, the compromised T-cell responses induced by hCAF-DCs were further restored (Figure 4). In addition, when S31–201 and 1-MT were added together, this immunosuppressive effect was nearly inhibited totally (Figure 4). In these results, S31–201 produced better effects than 1-MT, perhaps because S31–201 targets an upstream regulator of IDO, leading to a significantly and persistently decreased IDO levels in the MLR co-culture system. Therefore, these results indicated that IDO secretion by hCAF-DCs was mediated by STAT3 activation.

### IL-6 is responsible for STAT3 activation and IDO production in hCAF-DCs

Next, we wondered how DCs were educated by hCAFs and the mechanisms involved in STAT3 and IDO activation. IL-6 is widely known as a classic regulator of STAT3 pathway. Notably, our western blotting results also showed that IL-6 was the key factor that mediated STAT3 activation and IDO secretion. As shown in Figure 5a, when IL-6 (50 ng/ml) was added, STAT3 phosphorylation levels as well as IDO production in the normal DCs were significantly upregulated. And after treatment of NA IL-6 (2 μg/ml) during a co-culture, hCAF-DC STAT3 activation and IDO expression were distinctly suppressed. Given that SDF-1α secreted by hCAFs could recruit DCs effectively, we also detected its effect on STAT3/IDO activation. However, SDF-1α did not impact this pathway in these western blotting results. T-cell proliferation during the MLR, as determined by flow cytometry in Figure 5b, further confirmed that IL-6 was responsible for inducing the immunosuppressive effects of hCAF-DCs. In addition, NA IL-6 in the co-culture system between hCAFs and DCs rescued the impaired T-cell proliferation. Therefore, our data proved that hCAFs are capable of converting normal DCs into IDO-producing cells through STAT3 activation mediated by IL-6.

## Discussion

Although it is known that CAFs possess the capacity to induce immunomodulation, how they impact the DC function remains unclear. In the present study, we observed for the first time that hCAFs recruit DCs and educate them to acquire a tolerogenic phenotype through IL-6-mediated STAT3 activation.

Here, monocyte-derived DCs were treated by hCAFs using a cell-to-cell interaction model *in vitro*. We found that hCAF-DCs expressed the human DC markers like CD1a, CD83 and HLA-DR, as well as the monocyte marker CD14 and the Treg-associated marker CTLA-4. It has been reported that there is a new population of CD14^+^CTLA-4^+^ regulatory DCs in the PBMCs of HCC patients that suppressed T-cell response via CTLA-4-dependent IL-10 and IDO production.^15^ However, how CD14^+^CTLA-4^+^ DCs are induced remains unknown. Consistent with this, we found that CD14^+^CTLA-4^+^ hCAF-DCs also inhibited T cell's function through IDO secretion. Our results might partly explain the source of CD14^+^CTLA-4^+^ regulatory DCs. Nevertheless, how CTLA-4 might act in hCAF-DCs still needs to be defined.

We further discovered that hCAF-DCs had an immunosuppressive effect on T-cell responses via IDO secretion. It has been proven that, as a rate-limiting enzyme in the tryptophan catabolism, IDO is involved with immune tolerance and immunosuppression in cancer due to its capacity to induce T-cell anergy and Treg expansion.^25, 26^ IDO can be produced by tumor cells themselves; however, more importantly, it is mainly expressed by tumor-recruited immune cells, particularly DCs.^27^ Many studies revealed that the regulatory DCs found in the tumor region highly express IDO, which helps to inhibit the antitumor immune responses.^28, 29^ However, the mechanisms by which IDO production is regulated in DCs have not been well established. It was reported that the IDO pathway in regulatory DCs is stimulated by vitamin D3 or prostaglandin E2 treatment.^30, 31^ And it was also found that IDO transcription is regulated by STAT3 activation.^23^ Consistent with these findings, we also report here that hCAF-DCs can release IDO and impair T-cell responses, which indicates that hCAFs possess a great ability to convert normal DCs into regulatory DCs. Therefore, our results proved that regulatory DCs could be induced not only by tumor cells, but also by tumor nonparenchymal cells. In addition, our data will contribute to a better understanding of the mechanisms involved in the generation of regulatory DCs in cancer patients.

STAT3 activation has been found to be responsible for tumor immune escape. A large body of evidence has showed that STAT3 is activated in both cancer and immune cells in many cancers.^32^ It was reported that STAT3 activation was essential for Th17 cell differentiation, which was blocked by STAT3 ablation in CD4^+^ cells.^33^ STAT3 was found to enhance Foxp3 expression by tumor-infiltrating Tregs and maintained the suppressive functions of Tregs.^34, 35^ Disruption of STAT3 signaling helped macrophages to restore an activated phenotype and the capacity to produce inflammatory cytokines in response to LPS.^36^ Phosphorylated STAT3 levels were also found to be upregulated within MDSCs from tumor-bearing mice^37^ and DC functions were enhanced in tumor-bearing mice that lacked STAT3 expression.^38^ In our study, we confirmed that hCAF-DC IDO secretion was regulated by the STAT3 pathway. This result further demonstrated that STAT3 was the key factor in controlling tumor immune escape. At the same time, STAT3 is also involved in cancer cells functions, thus making it a promising target for cancer immunotherapy.

In tumors, this wide activation of STAT3 in DCs was proven to be induced by IL-6 from tumor cells.^39^ Here, we found that hCAFs also shared this ability to produce IL-6 and upregulate the STAT3 phosphorylation levels in DCs, suggesting that hCAFs should not be ignored in inducing suppressive immune cells. In our study, when treated by IL-6, DCs upregulated STAT3 activation and produced IDO, leading to the immunosuppressive effects on T-cell responses. Moreover, in a previous study by our group, IL-6-treated DCs downregulated CD1a, CD83, CD80, CD86 and HLA-DR expression, and upregulated CD14 expression.^40^ Our results fully confirmed that IL-6 derived from hCAFs was responsible for inducing regulatory DCs.

In summary, our results are the first to fully prove that CAFs in HCC recruit DCs and educate them into regulatory DCs through IL-6-mediated STAT3 activation. Our findings provide important new insights into the mechanisms by which CAFs perform an immunosuppressive role in tumors by inducing regulatory DCs. These data will help us to determine the immunoregulatory effects of the tumor stroma and to improve immune-based anti-cancer therapies.

## Materials and methods

### Fibroblasts isolation and culture

Tumor samples from HCC patients, which were proven to be pathological, were obtained from Sun Yat-sen University Third Affiliated Hospital in Guangzhou, China. Samples were anonymously coded in accordance with local ethical guidelines (as stipulated by the Declaration of Helsinki), and written informed consent was obtained from the patients and healthy volunteers. Our study was approved by the Clinical Research Ethics Committee of the Sun Yat-sen University Third Affiliated Hospital. The hCAFs and fibroblasts obtained from foreskin as NFs were isolated and cultured as described previously,^1^ and were used within 3–10 passages in the subsequent experiments.

### Human CD14^+^ monocyte differentiation

CD14^+^ monocytes were isolated from healthy blood by Ficoll-Paque (1.077 g/ml; Invitrogen, Carlsbad, CA, USA) density-gradient centrifugation and MACS Monocyte Isolation Kit (Miltenyi Biotec, Bergisch Gladbach, Germany). In total, 10^6^ cells/ml were cultured in 3 ml of Roswell Park Memorial Institute 1640 medium with 10% fetal bovine serum in six-well flat-bottom plates. Recombinant granulocyte-macrophage colony-stimulating factor (50 ng/ml, PeproTech, Rocky Hill, NJ, USA) and rIL-4 (50 ng/ml, PeproTech) were added to the cultures every 3 days. At day 6, immature DCs were harvested and co-cultured with hCAFs or NFs. For the preparation of mDC, hCAF-mDCs (hCAF-DC) or NF-mDCs (NF-DC), these cells were further stimulated with LPS (100 ng/ml, Sigma-Aldrich, Saint Louis, MO, USA) for 3 days.

### Co-culture experiment

DCs were seeded onto hCAFs or NFs (10^5^ cells/well) at a density of 10^6^ cells per 3 ml per well in six-well plates. The cells were co-cultured in Roswell Park Memorial Institute 1640 medium with 10% fetal bovine serum for 3 days. To induce DC maturation, LPS (100 ng/ml) was added for another 3 days. After the LPS treatment, the hCAF-DCs (or NF-DCs) were separated by collecting the suspended cells. Neutralizing IL-6 monoclonal antibodies (NA IL-6) (2 μg/ml, R&D Systems, Minneapolis, MN, USA) and/or neutralizing SDF-1α monoclonal antibodies (2 μg/ml, R&D Systems) were added at day 0, 3 and 5 during the co-culture period.

### Flow cytometry

The Abs used for flow cytometry included fluorescein isothiocyanate-, phycoerythrin- or allophycocyanin-conjugated mouse anti-human CD3, CD4, CD8, CD1a, CD83, CD80, CD86, HLA-DR, CD14, CTLA-4, CD90, CD166 and CD44. All antibodies were purchased from eBioscience (San Diego, CA, USA), except CTLA-4, which was purchased from BioLegend (San Diego, CA, USA). The DCs were collected, washed twice and resuspended in 100 μl of phosphate-buffered saline containing 0.1% bovine serum albumin. After staining with either specific Abs or the appropriate isotype controls, the cells were incubated on ice for 30 min. Then, the cells were washed with phosphate-buffered saline containing 0.1% NaN3 and 0.5% bovine serum albumin, after which they were fixed with a 1% paraformaldehyde solution. The analyses were performed by FACScan using CellQuest software (BD Bioscience, Franklin Lakes, NJ, USA). For the intracellular staining of T cells, cell stimulation cocktail (2 μl/ml; eBioScience) was added during the last 10 h. Then, the cells were collected, washed twice and stained with monoclonal antibodies that were specific for CD4 or CD8 for 30 min at 4 °C. After a phosphate-buffered saline wash, the cells were fixed and permeabilized with fixation/permeabilization solution (eBioScience) for 15 min at room temperature. After another wash, the cells were stained with monoclonal antibodies against IFN-γ and IL-10 (eBioScience) for 20 min at room temperature. For the Treg staining, the T cells were prepared with the Treg staining Kit (eBioScience).

### Cytokine assays

The supernatants were harvested for IL-12p70, TGF-β, IL-10, HGF and TNF-β concentration measurements with the Bio-Plex Suspension Array System (Hercules, CA, USA).

### Migration assay

Cell migration assays were carried out in 24-well Transwell chambers (Corning Costar, New York, NY, USA) using polycarbonate membranes (5 μm pore size). Media alone, media with 10% fetal bovine serum, hCAF-conditioned medium, media with IL-6 or SDF-1α, hCAF-conditioned medium with NA IL-6 or neutralizing SDF-1α monoclonal antibodies were added into the lower chambers. The DCs were labeled with 10 μM carboxy fluoroscein succinimidyl ester (Invitrogen) in 1 ml phosphate-buffered saline at a density of 2 × 10^5^ cells for 5 min at room temperature and then added into the upper chamber. The cells were incubated at 37 °C with 5% CO_2_ for 6 h. Then, the cell number in the lower chambers was determined with an inverted fluorescence microscope.

### Mixed lymphocyte reaction

In T-cell proliferation assays, DCs were cultured in Roswell Park Memorial Institute 1640 medium supplemented with 10% fetal bovine serum at a concentration of 2 × 10^4^ cells per 200 μl per well in 96-well U-bottom plates containing 10^5^ allogeneic peripheral blood lymphocytes labeled with carboxy fluoroscein succinimidyl ester for 3 days. And anti-human CD3 (2 μl/ml) and CD28 (1 μl/ml) functional grade purified (eBioscience) were added. Then, the cells were harvested and analyzed by fluorescence-activated cell sorting. For the assays of intracellular staining of T cells and Treg staining, DCs were cultured with non-treated peripheral blood lymphocytes at the same density as mentioned above for 5 days.

### Western blot

Proteins were separated by 10% sodium dodecyl sulphate-polyacrylamide gel electrophoresis, immunoblotted with an Ab against phospho-STAT3, STAT3 (Cell Signaling Technology, Danvers, Mass, USA), IDO and β-actin (Santa Cruz, Dallas, TX, USA) and then visualized by enhanced chemiluminescence (Millipore, Billerica, MA, USA). The results were recorded using Kodak film.

### Statistical analysis

The data were analyzed by analysis of variance with Bonferroni's *post hoc* test for multiple comparisons. All *P*-values ⩽0.05 were considered statistically significant. *P⩽0.05, **P⩽0.01, ***P⩽0.001, ns, not significant. All statistical analyses were conducted using the Statistical Program for Social Sciences 17.0 software program (SPSS Inc., Chicago, IL, USA).
