# Supplementary figures and images for: Hepatic carcinoma-associated fibroblasts induce IDO-producing regulatory dendritic cells through IL-6-mediated STAT3 activation
Source: Oncogenesis. 2016 Feb 22;5(2):e198–. doi: 10.1038/oncsis.2016.7 (PMC5154347; doi:10.1038/oncsis.2016.7)

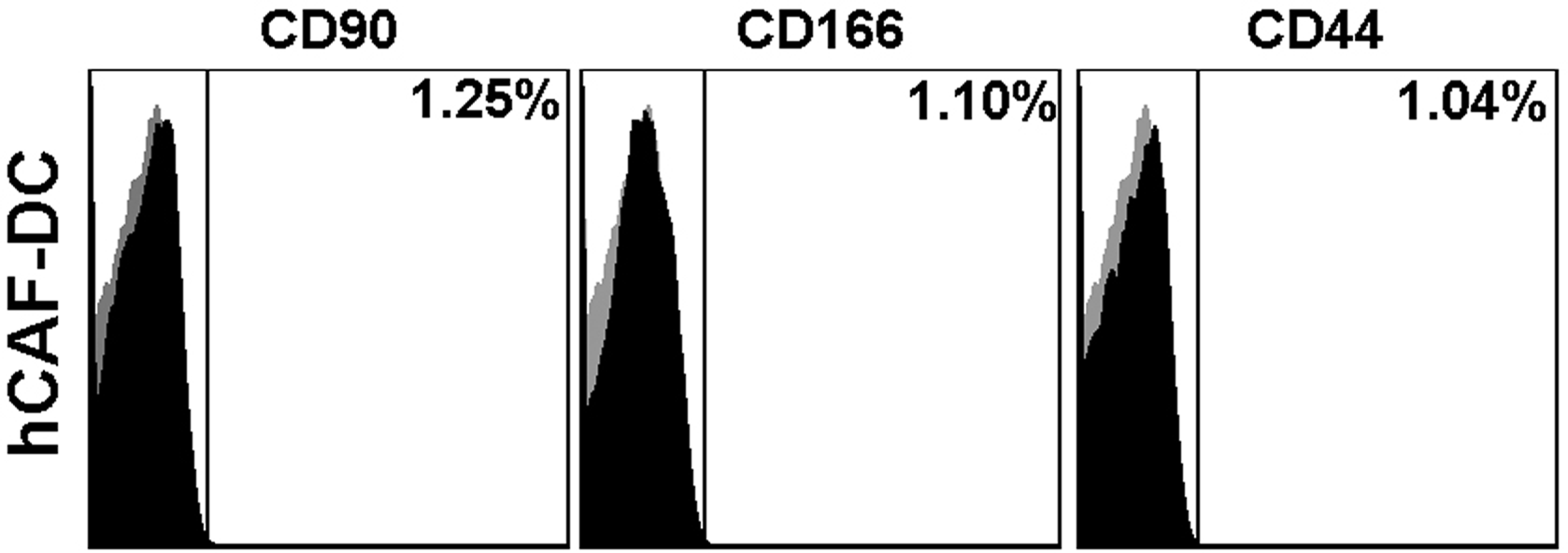

Supplement: Supplementary Figure 1 [file oncsis20167x1.tif]

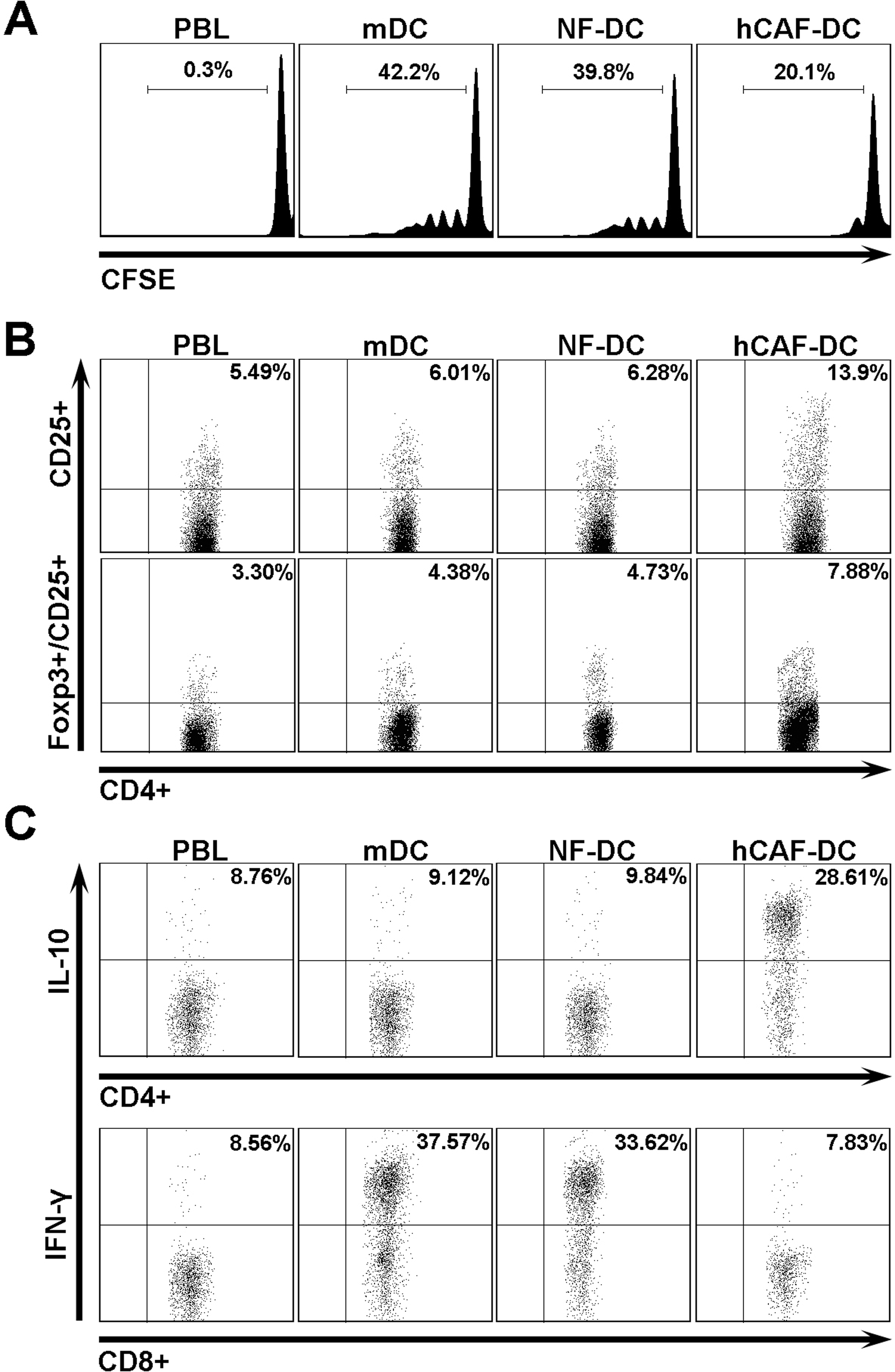

Supplement: Supplementary Figure 2 [file oncsis20167x2.tif]
