# Supplementary material for: Hepatic carcinoma-associated fibroblasts induce IDO-producing regulatory dendritic cells through IL-6-mediated STAT3 activation
Source: Oncogenesis. 2016 Feb 22;5(2):e198–. doi: 10.1038/oncsis.2016.7 (PMC5154347; doi:10.1038/oncsis.2016.7)
Supplement: Supplementary Figure Legends [file oncsis20167x3.doc]

**Figure S1. Immunophenotype analysis of hCAF-DCs.**

For excluding the contamination of hCAFs, CD90, CD166 and CD44 expression of hCAF-DCs was assessed by flow cytometry. One representative experiment of five is shown.

**Figure S2. hCAF-DCs exhibit tolerogenic characteristics.**

(A) Proliferation of CFSE-labeled PBLs was determined by flow cytometry after culturing alone, or co-culturing with mDCs, NF-DCs or hCAF-DCs for 5 days. One representative experiment of five is shown. (B) The effect of mDCs, NF-DCs or hCAF-DCs on CD4+CD25+ T cell and CD4+CD25+Foxp3+ Treg differentiation was assessed by flow cytometry. One representative experiment of five is shown. (C) IL-10 expression in CD4+ T cell, and IFN-γ expression in CD8+ T cell was detected by intracellular staining. One representative experiment of five is shown.
